# Enhancer-associated regulatory network and gene signature based on transcriptome and methylation data to predict the survival of patients with lung adenocarcinoma

**DOI:** 10.3389/fgene.2022.1008602

**Published:** 2022-09-23

**Authors:** Shihao Huang, Shiyu Chen, Di Zhang, Jiamei Gao, Linhua Liu

**Affiliations:** ^1^ Department of Biochemistry, Institute of Glycobiology, Dalian Medical University, Dalian, Liaoning, China; ^2^ Department of Laboratory Medicine, Nanxishan Hospital of Guangxi Zhuang Autonomous Region, Guilin, China

**Keywords:** lung adenocarcinoma, enhancer, methylation, regulatory network, gene signature, prognostic model

## Abstract

Accumulating evidence has proved that aberrant methylation of enhancers plays regulatory roles in gene expression for various cancers including lung adenocarcinoma (LUAD). In this study, the transcriptome and methylation data of The Cancer Genome Atlas (TCGA)-LUAD cohort were comprehensively analyzed with a five-step Enhancer Linking by Methylation/Expression Relationships (ELMER) process. Step 1: 131,371 distal (2 kb upstream from the transcription start site) probes were obtained. Step 2: 10,665 distal hypomethylated probes were identified in an unsupervised mode with the get.diff.meth function. Step 3: 699 probe-gene pairs with negative correlations were screened using the get.pair function in an unsupervised mode. Step 4: After mapping with probes, 768 motifs were obtained and 24 of them were enriched. Step 5: 127 transcription factors (TFs) with differential expressions and negative correlations with methylation levels were screened, which were corresponding to 21 motifs. After the ELMER process, a prognostic “TFs-motifs-genes” regulatory network was constructed. The Least absolute shrinkage and selection operator (LASSO) and Stepwise regression analyses were further applied to identify variables in the TCGA-LUAD cohort and an eight-gene signature was constructed for calculating the risk score. The risk score was verified in two independent validation cohorts. The area under curve values of receiver operating characteristic curves predicting 1-, 3-, and 5-years survival ranged from 0.633 to 0.764. With the increase of the risk scores, both the survival statuses and clinical traits showed a worse tendency. There were significant differences in the degrees of immune cell infiltration, TMB values, and TIDE scores between the high-risk and low-risk groups. Finally, a better-performing prognostic nomogram was integrated with the risk score and other clinical traits. In short, this multi-omics analysis demonstrated the application of ELMER in analyzing enhancer-associated regulatory network in LUAD, which provided promising strategies for epigenetic therapy and prognostic biomarkers.

## Introduction

Enhancer is a DNA sequence in the genome with a length of 50–1,500 bp, which can bind with transcription factors (TFs) to promote the transcription of the target gene. The position of enhancer is not fixed and can be at the near end or the far end of a target gene. The enhancer may be upstream or downstream of its regulatory gene (Field and Adelman, 2020).

Enhancers have been reported to reflect normal and pathogenic cellular conditions (Fishilevich et al., 2017). Some high-throughput identification approaches have been developed to predict the enhancers and corresponding functions (Kleftogiannis et al., 2016). With the progress of functions, enhancers have been found to link with several diseases (Wang et al., 2018). Researchers have tried to build a database for disease-associated enhancers: DiseaseEnhancer (Zhang et al., 2018).

Reviews are describing the roles of enhancers in tumors (Sur and Taipale, 2016). As important regulatory elements of DNA, enhancers participate in several comprehensive regulatory networks of cancer-associated genes. Mutations in tumors often lead to aberrantly regulated enhancers, as well as abnormal expression of growth-related genes (Adhikary et al., 2021). The abnormal regulation can be *trans*-action, such as the activation of a transcription factor or apparent regulatory factors that control enhancer activity. Similarly, abnormal regulation can also be *cis*-action, such as mutation to change enhancer activity or its specificity to the target gene (Chen and Liang, 2020). Investigating the activity regulation and related mechanism of tumor type-specific enhancers at the molecular level may be applied for screening therapeutic targets.

Accumulating evidence has suggested that many aberrant methylation sites have been observed on enhancer sequences in cancer cells (Herz, 2016). These abnormal methylations have been proven to link with the expression of a target gene, as well as the disease progression. The methylation state of enhancer regions is the promising next generation of epigenetic biomarkers (Clermont et al., 2016). One study has revealed the abnormal enhancer of hepatocellular carcinoma (HCC) based on multi-omics data (Xiong et al., 2019). By comprehensive analysis of ChIP-seq data, transcriptome data, DNA methylation data, and HiC data, the abnormal enhancer and related transcription disorders in HCC have been described, and the differentially methylated enhancer and its target genes were identified. A prognostic model based on these differentially expressed genes (DEGs) of abnormal enhancers was constructed, which predicted the prognosis of HCC. It was beneficial to the development of epigenetic therapy for HCC (Huang et al., 2022). However, the epigenetic regulation and function of transcription enhancers have been still unclear.

Lung cancer is the leading cause of cancer mortality. It is classified into various histologic subtypes, including adenocarcinoma, squamous carcinoma, non-small cell lung cancer, and small cell lung cancer (Ruiz-Cordero and Devine, 2020). Lung cancer exhibited a good response to novel targeted therapies, such as checkpoint immunotherapy. With the advances in knowledge on molecular characteristics of lung cancer, researchers have found different treatment decisions should be provided to patients with varied gene expression profiles (Chen et al., 2020). For example, some patients may show a better response to immunotherapy, while others should receive targeted therapies and chemotherapy before considering immunotherapy as a single agent (Mazieres et al., 2019). Drug resistance should also be considered (Denisenko et al., 2018). Some studies have tried to find the diagnostic and prognostic prediction markers for lung cancers, such as the immune-related genes signature (Yi et al., 2021), m6A modification (Li et al., 2021), and costimulatory molecule-based signature (Zhang et al., 2020). All these efforts have contributed to the precision and individual treatment of patients with tumors.

**FIGURE 1:**
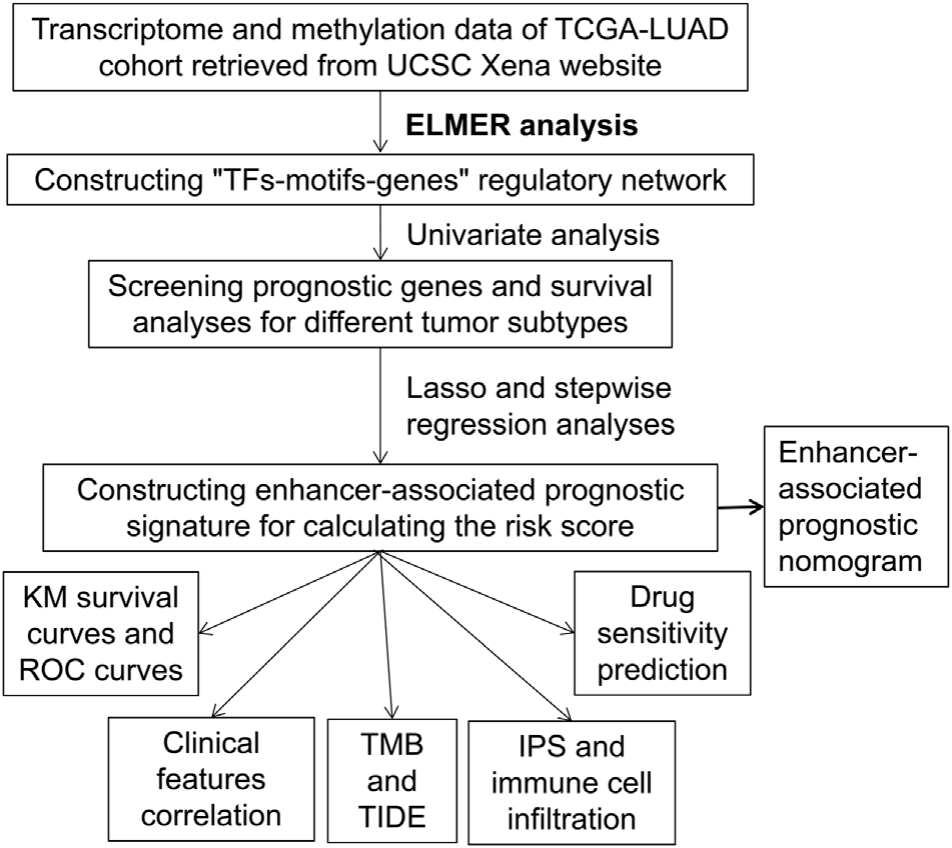
The workflow for obtaining enhancer-associated prognostic signature.

This study aims to find enhancer-associated prognostic biomarkers. The transcriptome and methylation of lung adenocarcinoma (LUAD) cohorts have been integrated. The enhancer-associated regulatory network was constructed after Enhancer Linking by Methylation/Expression Relationships (ELMER) analysis. An enhancer-associated prognostic gene signature has been constructed with the screened target transcription factor and target genes, exhibiting good prognostic prediction performance for patients with lung cancers. The complete analysis route of this study has been provided in Figure 1.

## Materials and methods

### Datasets

Three cohorts were applied: The Cancer Genome Atlas (TCGA)-LUAD, GSE31210, and GSE8894. Both the transcriptome and methylation data of TCGA-LUAD were retrieved from the UCSC Xena website, and the transcriptome data of GSE31210 and GSE8894 were obtained from the Gene Expression Omnibus (GEO) database. The clinical features were also obtained, and the data from patients with overall survival of <30 days were removed. All transcriptomic data were normalized with log_2_ (x+1) method and the combat function of the sva R package was used to exclude batch effects (Leek et al., 2012). The methylation data were normalized with the champ.norm function of the ChAMP R package, and the missing values in methylation data have been filled with function impute.knn (Tian et al., 2017).

### Enhancer-associated regulatory network

With the gene methylation and transcription levels of the same batch of samples in the TCGA-LUAD cohort, the sequences of the differential methylation probes were screened with the ELMER R package (Silva et al., 2019), found the enriched motifs, and further predicted the TFs interacting with these motifs. Finally, the “TFs-motifs-genes” regulatory network was constructed. ELMER’s main analysis process consisted of the following five parts: 1) Identification of distal probes (probes larger than 2 kb upstream from the transcription start site) from methylation chip data; 2) identification of differences in methylation levels between normal and tumor groups; 3) identification of target genes for differentially methylated probes; 4) identification of motifs enriched with both differentially methylated and target gene-related probes; 5) identification of TFs based on transcriptional differences.

### Identification of prognostic regulatory network and tumor subtypes

Based on the transcriptional level of genes included in the regulatory network, we first conducted the univariate analysis to screen prognostic genes, and then performed the unsupervised clustering in the three cohorts with the ConsensusClusterPlus R package by the k-means method (Wilkerson and Hayes, 2010). The clustering process was carried out 1,000 times, involving 80% samples in each iteration. Subsequently, survival analyses were performed for different subtypes.

### Construction of enhancer-associated prognostic signature

TCGA-LUAD was applied as the training set, GSE31210 and GSE8894 were applied as the validation sets. Least absolute shrinkage and selection operator (LASSO) and Stepwise regression analyses were applied to further streamline prognostic variables in the TCGA-LUAD cohort and construct a multi-gene COX signature for calculating the risk score of each patient (Tibshirani, 1997). Patients were divided into high-risk and low-risk groups based on the median risk score. Then Kaplan–Meier (KM) survival curves and Receiver Operating Characteristic (ROC) curves were plotted to assess the prediction effect of the model. Independent prognostic analyses were applied for validating the independence of the risk score compared with other clinical features in the three cohorts. The Wilcoxon rank sum test was applied to evaluate correlations between risk score and clinical features in TCGA-LUAD cohort.

Analysis of tumor mutation burden (TMB), tumor immune dysfunction and exclusion (TIDE), and immune micro-environment.

The mutation data of TCGA-LUAD based on VarScan2 (Koboldt et al., 2012) were obtained from TCGA Database. Non-synonymous mutations were calculated and the TMB scores were obtained with the number of variants/the length of exons. Wilcoxon test was used to analyze the difference in TMB values. The Maftools R package was used to calculate and plot the somatic alterations landscapes (Mayakonda et al., 2018). The TIDE scores of the TCGA-LUAD cohort were obtained from TIDE (Jiang et al., 2018). The differences in the immune micro-environment were compared with the proportions of 22 types of immune cells estimated by Cibersort (Chen et al., 2018). The Cibersort R package was applied. The simulation was conducted 1,000 times with the parameter of perm = 1,000, QN = True. The samples with *p* > 0.05 were rejected and removed. The correlation between risk scores and various immune cell infiltration was further analyzed with the Spearman correlation test.

### Analysis of tumor immunogenicity and drug susceptibility

The tumor immunogenicity was analyzed in patients from high-risk and low-risk groups, which was divided by the median value of the risk score. The immunophenotype scores (IPS) of TCGA-LUAD cohort were downloaded from The Cancer Immunome Atlas database (https://tcia.at/home). Based on the expression status of CTLA4 and PD1 (Charoentong et al., 2017), the high-risk and low-risk groups were further classified into four subgroups: positive CTLA4 and positive PD1; positive CTLA4 and negative PD1; negative CTLA4 and positive PD1; negative CTLA4 and negative PD1. The Wilcoxon nonparametric test was used to compare the differences in IPS between high-risk and low-risk groups in each subgroup. The drug susceptibility has been explored in patients from high-risk and low-risk groups. The pRRophetic R package was applied to analyze IC50 values of six commonly used drugs (Cisplatin, Docetaxel, Erlotinib, Gefitinib, Gemcitabine, and Paclitaxel).

### Integration of the enhancer-associated prognostic nomogram

The predictive efficacy of the risk score for other clinical symptoms was assessed by ROC curves for 1, 3, and 5 years of survival. The risk score and clinical features (Gender, Age, Stage, Prior-malignancy) were integrated into a nomogram using the “rms” R package. The ROC curves of the nomogram and clinical features for the 5-year survival were plotted with the survivalROC R package. The performance of the nomogram was also confirmed by both the KM and calibration curves.

### Statistics

Statistical analysis was conducted with the R 4.0.3. The survival analyses were performed with the log-rank test. The comparison between the two groups was executed with the Wilcoxon test or *t*-test. *p* < 0.05 was considered to be significant.

## Results

### Construction of enhancer-associated regulatory network with ELMER

**FIGURE 2:**
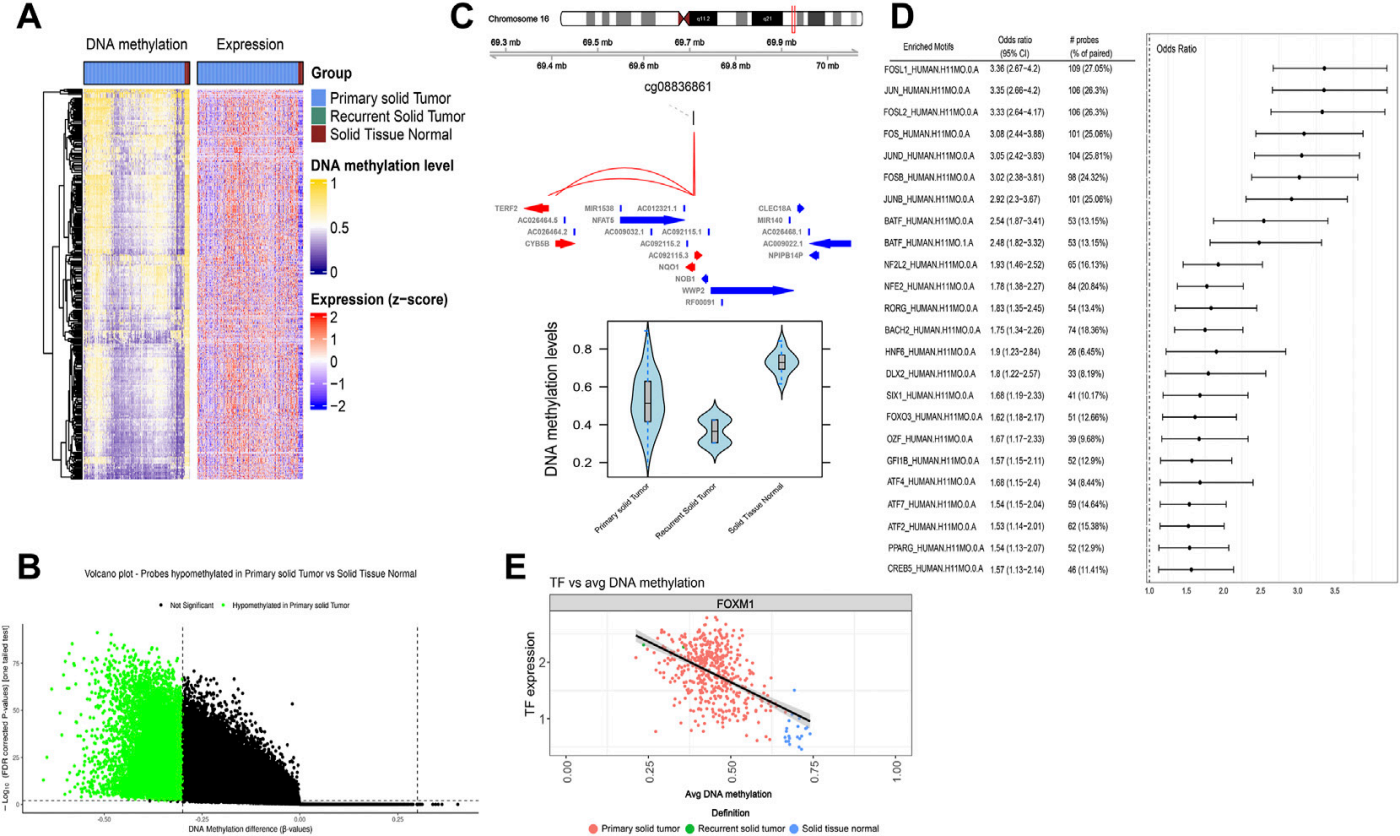
Enhancer-associated regulatory network was constructed with ELMER. **(A)** The heatmap of methylation and transcriptomic data in the TCGA-LUAD cohort. **(B)** Volcano plot of probes hypomethylated in primary tumor tissues. **(C)** Example of top ten genes closest to the upstream and downstream of the differentially methylated distal probes. **(D)** Odds ratios of the significantly enriched motifs identified by the get.enriched.motif function. **(E)** Example of correlation plot between the TF expression level and corresponding average DNA methylation level.

Both methylation and transcriptomic data of 463 primary tumor tissues and 21 normal controls from the TCGA-LUAD cohort were fed into subsequent ELMER analysis. The heatmap of methylation and transcriptomic data is plotted (Figure 2A). Distal probes are the region where enhancers are enriched. Based on the hg38 reference genome file, we first select 13,1371 distal probes with the get.feature.probe function (Supplementary Material S1). Then, the unsupervised mode is used to identify distal hypomethylated probes with the get.diff.meth function. In detail, for every distal probe, the methylation levels are sorted in all samples within the primary tumor and the normal groups separately, and those samples in the lower quintile (20% samples with the lowest methylation levels) of each group are used to identify whether the probe is hypomethylated in the tumor group, thus obtaining 10,665 distal hypomethylated probes (Supplementary Material S2) with the threshold of false discovery rate (FDR) < 0.01 and Δβ < −0.3 (Figure 2B). Next, the GetNearGenes function is applied to identify the top ten genes closest to the upstream and downstream of the distal hypomethylated probes separately, generating probe-gene pairs (Figure 2C). Then, for each probe-gene pair, the inverse correlations between the methylation level of the probe and the expression of the gene were tested. The top 20% and the bottom 20% of all the samples based on the probe’s methylation level are extracted as the Methylated (M) group and Unmethylated (U) group. The gene expression levels between M and U groups are compared by the Mann-Whitney *U* test. 669 pairs of statistically significant probe-gene pairs with negative correlations are screened by default parameters using the get.pair function in an unsupervised mode (Supplementary Material S3). Further, the 250 bp base sequence upstream and downstream of the probes screened in the previous step are extracted, mapped to 768 motifs (Supplementary Material S4), and identified 24 significantly enriched motifs by the get.enriched.motif function (Figure 2D). Finally, based on the methylation level, the distal probes corresponding to the same motif were classified as the top 20% M group and the bottom 20% U group. A total of 127 TFs (Lambert et al., 2018) with differential expressions in the two groups and negative correlations with methylation levels are screened by using the get.TFs function in an unsupervised mode (Figure 2E), corresponding to 21 motifs (Supplementary Material S5).

### Construction of prognostic network and its contribution to tumor subtypes

**FIGURE 3:**
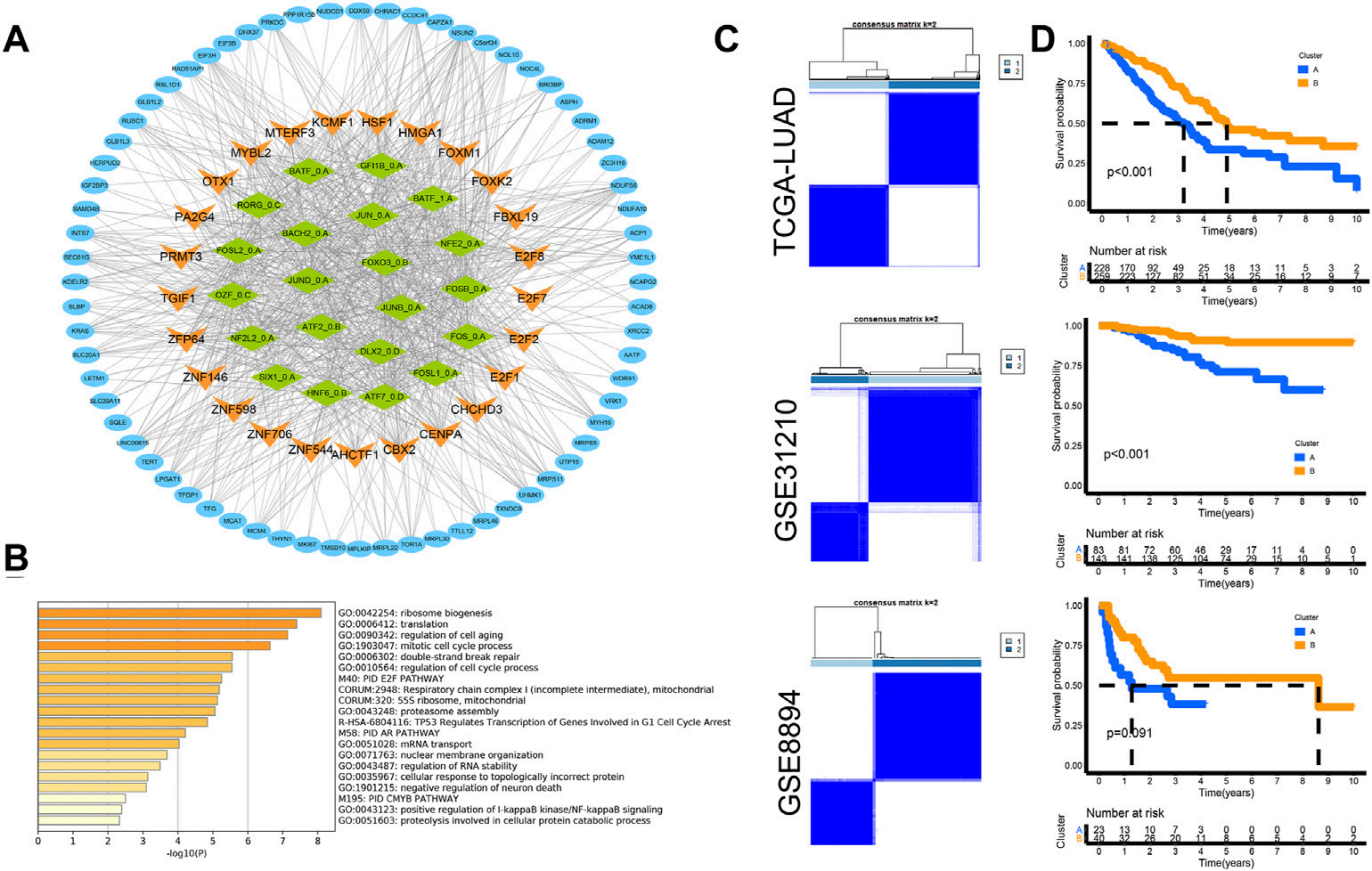
Prognostic network was constructed and contributed to tumor subtypes. **(A)** Prognostic regulatory network visualized with the Cytoscape software. **(B)** Function enrichment performed by Metascape. **(C)** Heatmaps of unsupervised consensus matrixes in the three cohorts. **(D)** KM curves revealed significant survival differences in all three cohorts. Log-rank test.

With the 21 motifs as links, 127 TFs and 271 target genes are screened in the enhancer-associated regulatory network. According to expression data in the TCGA-LUAD cohort, 25 TFs and 80 target genes are selected as prognostic genes with the univariate analysis *p* < 0.05 (Supplementary Material S6). Then, the prognostic regulatory network is visualized with the Cytoscape software (Figure 3A). The function enrichment is performed with the Metascape (Zhou et al., 2019) webtool (https://metascape.org/). The results show that the regulatory network mainly affected ribosome biogenesis, translation, cell aging, cell cycle, E2F pathway, and so on (Figure 3B). Then, based on the transcriptional data of the regulatory network, unsupervised clustering analysis is conducted in the three cohorts respectively. Two stable subtypes are obtained (Figure 3C), and the KM curves reveal significant survival differences (Figure 3D).

### An eight-gene enhancer-associated prognostic signature

**FIGURE 4:**
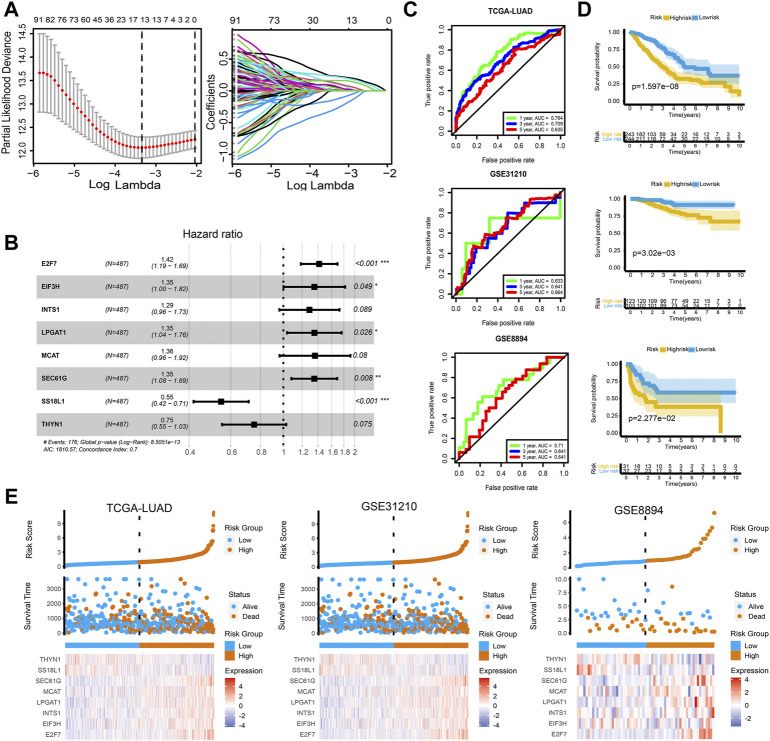
An eight-gene enhancer-associated prognostic signature. **(A)** Lasso regression analysis result. Partial Likelihood Deviance profile (left) and coefficients profile (right) changing with the log lambda. **(B)** Forest plot of each gene’s hazard ratio (HR) and the model’s concordance index. **(C)** ROC curves predicting 1-, 3-, and 5-years survival in the TCGA-LUAD, GSE31210, and GSE8894 cohorts. **(D)** KM curves showed that there were significant differences in survival between the high- and low-risk groups in all three cohorts. **(E)** The expression heatmaps of eight genes in the model and the patients’ risk factor correlation curves.

**FIGURE 5:**
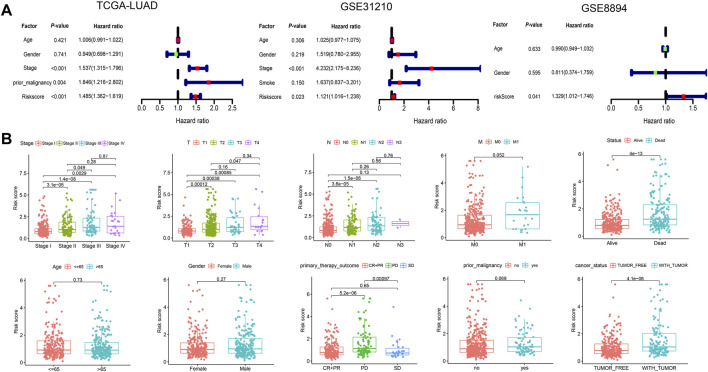
The risk score was an independent factor for survival. **(A)** Multi-cox analyses of the risk score and other clinical features in the three cohorts. **(B)** Differential analyses of the risk scores in patients with different clinical traits. With the increase of tumor stages and degrees of metastasis, the risk scores showed a gradually increasing trend, and the risk scores of patients with PD responses to the primary therapy were significantly improved compared to those with complete response (CR), partial response (PR), and stable disease (SD) responses.

Next, TCGA-LUAD is applied as the training set, with GSE31210 and GSE8894 as the validation sets. Relevant clinical features are presented (Table 1). With the 105 genes in the above regulatory network as the initial variables, we subsequently conduct Lasso regression analysis (Figure 4A) and Stepwise regression analysis, and finally obtain the Cox model consisting of genes. The risk score = (0.3486 × E2F7) + (0.3011 × EIF3H) + (0.2536 × INTS1) + (0.3019 × LPGAT1) + (0.3078 × MCAT) + (0.3006 × SEC61G) + (−0.6044 × SS18L1) + (−0.2837 × TNYN1). The forest plot shows the hazard ratio (HR) of each gene, and the model’s concordance index reached 0.7 (Figure 4B). Detailed results of variables within the model are shown in Table 2. The AUC of ROC curves for 1-, 3-, and 5-years survival are 0.764, 0.709, and 0.635 in the TCGA-LUAD cohort, and ranged from 0.633 to 0.71 in the independent validation cohorts GSE31210 and GSE8894 (Figure 4C). KM curves show significant differences between the high-risk and low-risk groups in all three cohorts (Figure 4D). The expression heatmaps of eight genes involved in the model and the risk curves of patients were also visualized. With the increase of the risk scores, the survival statuses become worse, and the expression levels of SS18L1 and THYN1 are gradually decreased, while the expression levels of the other six genes are gradually increased (Figure 4E). The results are consistent with the HR value of each gene in the model. Based on the results of multi-cox prognostic analyses, the risk score is an indicator independent of other factors (*p* < 0.05) for predicting the survival in all three cohorts (Figure 5A). Correlation analysis with clinical traits shows that with the increase of tumor stages and degrees of metastasis, the risk scores exhibit a gradually increasing trend, and the risk scores of patients with PD responses to the primary therapy exhibit significant improvement compared to those with complete response (CR), partial response (PR), and stable disease (SD) responses (Figure 5B).

**TABLE 1:** Clinical characteristics.

Feature	TCGA-LUAD (N = 487)	GSE31210 (N = 226)	GES8894 (N = 63)
Age			
>65	230 (47.23%)	50 (22.12%)	18 (28.57%)
≤65	247 (50.72%)	176 (77.88%)	43 (68.25%)
Unknown	10 (2.05%)	NA	2 (3.17%)
Gender			
Male	226 (46.41%)	105 (46.46%)	34 (53.97%)
Female	261 (53.59%)	121 (53.54%)	29 (46.03%)
Stage			
I	262 (53.80%)	168 (74.34%)	NA
II	114 (23.41%)	58 (25.66%)	NA
III	79 (16.22%)	NA	NA
IV	25 (5.13%)	NA	NA
Unknown	7 (0.01%)	NA	NA

NA: Not Available.

**TABLE 2:** Detailed results of variables within the enhancer-associated signature.

ID	Coef	HR	HR.95 L	HR.95H	*p*-value
E2F7	0.3486	1.4171	1.1862	1.6930	1.22E-04
EIF3H	0.3011	1.3514	1.0013	1.8239	4.90E-02
INTS1	0.2536	1.2886	0.9622	1.7258	8.89E-02
LPGAT1	0.3019	1.3524	1.0376	1.7627	2.55E-02
MCAT	0.3078	1.3604	0.9639	1.9200	7.99E-02
SEC61G	0.3006	1.3507	1.0811	1.6876	8.13E-03
SS18L1	-0.6044	0.5464	0.4179	0.7144	9.90E-06
THYN1	-0.2837	0.7530	0.5511	1.0287	7.47E-02

### Differences in TMB, TIDE, and immune micro-environment

TMB score represents the density of non-synonymous mutation distribution in the protein-coding region, which is calculated with the number of non-synonymous mutation sites/the total length of exons. TMB is an effective index to predict the response of immunotherapy, and higher TMB indicates a better immune response. TIDE is the computational algorithm for assessing tumor micro-environment from gene expression profiles. TIDE involves a set of gene expression markers to evaluate tumor immune evasion, including dysfunction of tumor-infiltrating cytotoxic T lymphocytes (CTL) and exclusion of CTL by immunosuppressive factors. TIDE is a quantitative index of immune escape, and higher TIDE indicates more serious the immune escape of the tumor.

**FIGURE 6:**
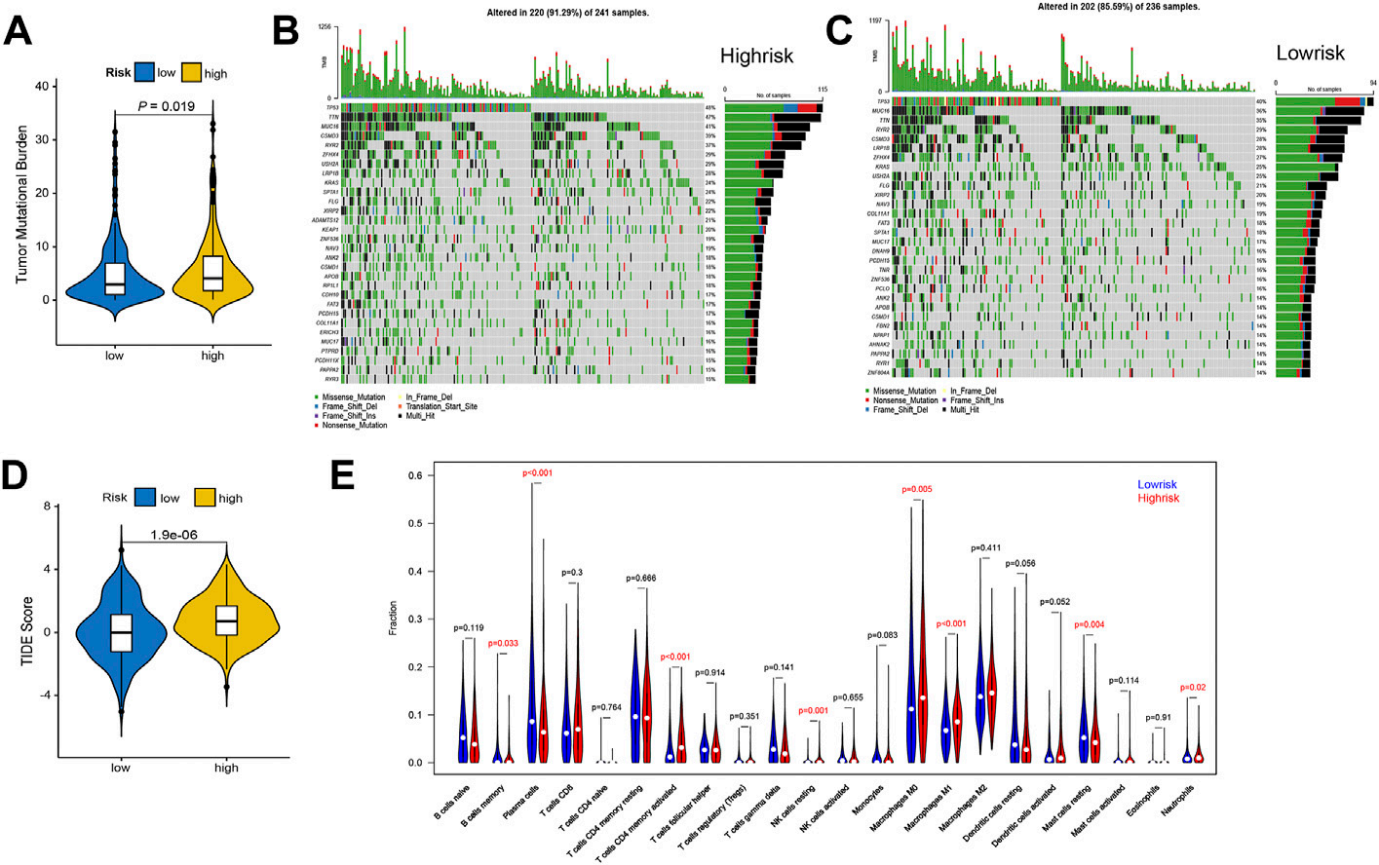
Differences of tumor mutation burden and immune micro-environment in high- and low-risk patients. **(A)** The violin diagram shows the TMB difference between the two groups. **(B)** Mutation profile of the top 30 genes with the biggest mutation frequency in high-risk samples. **(C)** Mutation landscape of the top 30 genes with the biggest mutation frequency in low-risk samples. **(D)** Violin diagram of the TIDE scores in the two groups. **(E)** The violin diagram of the differences between two groups in the infiltration of 22 immune cells.

**FIGURE 7:**
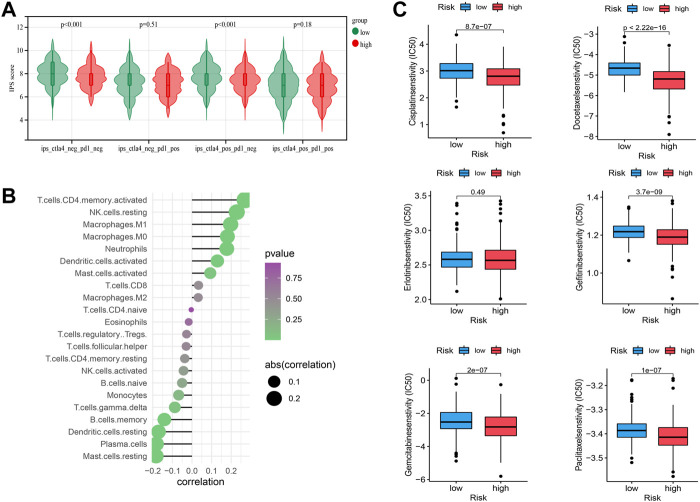
Tumor immunogenicity and drug susceptibility. **(A)** IPS score in low-risk and high-risk groups, which were classified into four subgroups: positive CTLA4 and positive PD1; positive CTLA4 and negative PD1; negative CTLA4 and positive PD1; negative CTLA4 and negative PD1, respectively. **(B)** The correlation between risk scores and various immune cell infiltration. **(C)** The IC50 values of Cisplatin, Docetaxel, Erlotinib, Gefitinib, Gemcitabine, and Paclitaxel in low-risk and high-risk groups.

To explore the characteristics of the immune microenvironment in patients with high and low risks, we compare the TMB values, TIDE scores, and 22 types of immune cell infiltration in these two groups. The high-risk group shows significantly higher TMB values (Figure 6A), and higher percentages of gene mutations (Figure 6B), compared to that of the low-risk group (Figure 6C). The high-risk group also presents higher TIDE scores than the low-risk group (Figure 6D). Eight of the 22 immune cell types show statistical differences in the infiltration degree. Among them, macrophage M0, macrophage M1, and T cell CD4 memory activated significantly increase in the high-risk group, while plasma cells and mast cells resting decrease significantly (Figure 6E). T cell CD4 memory activated exhibits a significant positive correlation with the risk score, while mast cells resting presents a significant negative correlation with the risk score (Figure 7B).

### Tumor immunogenicity and drug susceptibility

The IPS is included for evaluating tumor immunogenicity. Higher IPS suggested higher immunogenicity, exhibiting a potential higher response rate to immunotherapy. The IPS score of the low-risk group is significantly higher than that of the high-risk group in subgroups of positive CTLA4 and positive PD1, negative CTLA4 and negative PD1 (Figure 7A). The results suggest that the patients in the low-risk group show a better response to immunotherapy. The IC50 values of six commonly used drugs (Cisplatin, Docetaxel, Erlotinib, Gefitinib, Gemcitabine and Paclitaxel) are calculated in low-risk and high-risk groups. For all six drugs except for Erlotinib, LC50 is significantly higher in low-risk groups than that of high-risk groups (Figure 7C). It suggests that the patients in the high-risk group may be more sensitive to these drugs, which is accompanied by the high potential of immunosuppression.

### An integrated enhancer-associated prognostic nomogram

**FIGURE 8:**
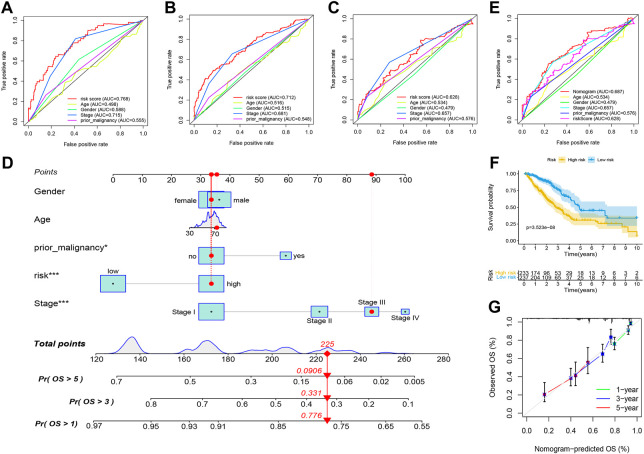
Integration of the enhancer-associated prognostic nomogram. **(A)** ROC analyses of the risk score with other clinical traits (Age, Gender, Stage, and Prior-malignancy status) in predicting 1-year survival. **(B)** ROC analyses of multiple indicators in predicting 3-years survival. **(C)** ROC analyses of multiple indicators in predicting 5-years survival. **(D)** Nomogram including all the variables in the TCGA-LUAD cohort integrated with multi-cox regression analysis. **(E)** ROC analyses of the nomogram with other clinical traits in predicting 5-years survival. **(F)** KM curve of the high- and low-risk patients distinguished by the nomogram. **(G)** Calibration curve of the nomogram for the survival prediction.

We first compare the predictive accuracy of the risk score with various clinical traits (Age, Gender, Stage, and Prior-malignancy status). ROC analyses of multiple indicators show that the risk score is more accurate compared to other clinical traits in predicting 1- and 3-years survival (Figure 6B–Figure 8A), and slightly lower than Stage in predicting 5-years survival (Figure 8C). Then, a nomogram including all the variables in the TCGA-LUAD cohort is integrated with multi-cox regression analysis (Figure 8D). The nomogram exhibited the highest accuracy in the ROC curve for predicting 5-years survival, higher than the risk score alone (Figure 8E). And the performance is also validated with the KM curve (Figure 8F) and the calibration curve (Figure 8G).

## Discussion

Lung cancer has been a common type of cancer (Mao et al., 2016). Its prognosis has been generally poor, especially for the advanced stages (Jones and Baldwin, 2018). As a huge burden on society (Bade and Dela Cruz, 2020; Wu et al., 2021), the high incidence and mortality of lung cancer put forward higher and more urgent demands for its early diagnosis and treatment (Rodriguez-Canales et al., 2016). With the development of molecular biology tools, the genomic information of lung cancer has been better profiled at the molecular level, which provided new treatment options and improved outcomes for patients (Parikh, 2019). Several studies have tried to find diagnostic and prognostic prediction markers for lung cancers. One prospective cohort study included 426 patients with complete surgical resection of stages I to III LUAD reported that a computational machine-learning prediction model integrating genomic and clinicopathologic features could better predict the risk of recurrence, compared with the current TNM system. It would provide recommendations for adjuvant therapy after surgical resection of LUAD (Jones et al., 2021). Another study performed on LUAD patients in eastern China detected molecular alterations with a customized DNA panel. Some of the specific mutations may make effects on the efficacy of targeted therapies, CCAAT enhancer binding protein alpha (*CEBPA*) mutations affected the efficacy of EGFR-tyrosine kinase inhibitors. The erb-b2 receptor tyrosine kinase 2 (*ERBB2*), CEBPA and transcription factor 7 like 2 (*TCF7L2*) mutated tumors tend to have higher TMB. The targeted DNA panel may be helpful for personalized treatment decisions of LUAD patients (Liu et al., 2021).

For better understanding the molecular characteristics of lung cancers, several studies have involved various bioinformatic tools to comprehensively analyze the omics data based on microarray or sequencing analysis of patients. Based on the microarray datasets of three cohorts of lung cancer, a meta-analysis has been performed. There were 50 upregulated and 87 downregulated genes overlapped in three datasets, which were included in following analysis. With the protein-protein interaction (PPI) networks, 22 core genes were identified, which were all significantly associated with poor survival. Finally, KEGG pathway enrichment reanalysis screened five key genes, which exhibit a relationship with certain drugs. The identified key genes can be candidate targets for both the treatment and prognosis of lung cancer (Wang et al., 2021). With the synthetic analysis of the transcriptome sequencing dataset and a non-coding RNA sequence dataset of small-cell lung cancer, the differentially expressed genes and miRNAs can be screened. After function enrichment, the molecular mechanisms were identified with the PPI network. Finally, 19 overlapping target genes and 32 corresponding regulatory miRNAs were screened. The bioinformatics analysis involving multi-omics data can assist in exploring the roles of target genes, miRNA, and TFs, which may better understand the potential molecular pathways (Mao et al., 2019).

After screening the potential candidate genes based on bioinformatics analysis, they can be further validated experimentally. A comprehensive bioinformatics analysis revealed that, the decreased expression of immunoglobulin superfamily member 10 (IGSF10) was associated with the shortened overall survival duration of patients with lung cancer. In subsequent experimental validation, IGSF10-knockout cells presented significantly increased proliferation and adhesion capability, revealed by MTT, colony formation assay, and Transwell assay, respectively. Further, Western blotting suggested that, the IGSF10-knockout can activate the integrin-β1/FAK pathway, presented as the upregulated protein expression levels of integrin-β1, phosphorylated (p)-FAK and p-AKT (Ling et al., 2020). Another study has analyzed the RNA sequencing data and revealed circXPO1, a novel circular RNA (circRNA) in LUAD. The circXPO1 was derived from a well-established cancer therapeutic target, XPO1, which was highly expressed in LUAD tissues compared with paired controls. High circXPO1 expression was correlated with worse overall survival. Mechanically, circXPO1 could bind with IGF2BP1 and enhance CTNNB1 mRNA stability, and subsequently promote LUAD progression (Huang et al., 2020). A similar study verified that circ-CAMK2A enhanced LUAD metastasis by regulating the miR-615-5p/fibronectin one pathway. Circ-CAMK2A upregulated the expression level of fibronectin one by sponging miR-615-5p, thus promoting MMP2 and MMP9 expression to stimulate the metastasis of LUAD (Du et al., 2019). In short, the combination of bioinformatics analysis and experimental verification can better clarify the significance of certain target biomarkers or promising pathways.

In our study, the comprehensive multi-omics analysis has also been applied to screen the target genes, which may be significant to prognosis prediction of lung cancer. The transcriptome and methylation data of the TCGA-LUAD cohort involving 463 primary tumor tissues and 21 normal controls were obtained for subsequent ELMER analysis. A total of 127 TFs corresponding to 21 motifs and 271 target genes were screened for constructing the subsequent enhancer-associated regulatory network. 25 TFs and 80 target genes were selected as prognostic genes with the univariate analysis *p* < 0.05. With TCGA-LUAD as the training set, the Cox model involving eight genes was selected with LASSO regression analysis and Stepwise regression analysis. The risk score = (0.3486 × E2F7) + (0.3011 × EIF3H) + (0.2536 × INTS1) + (0.3019 × LPGAT1) + (0.3078 × MCAT) + (0.3006 × SEC61G) + (−0.6044 × SS18L1) + (−0.2837 × TNYN1). With the increase of the risk scores, the survival statuses became worse, as well as the clinical traits including tumor stages, metastasis degree, and treatment responses. The risk score exhibited prognostic prediction accuracy with GSE31210 and GSE8894 as the validation sets.

The multi-omics study has been proved as a good tool for the epigenetic regulation of functional enhancers. In a study on HCC, methyl-binding DNA capture sequencing was firstly performed on both tumor and control tissues. The data revealed abnormal enhancer hypermethylation patterns. Then, the single-base resolution whole-genome bisulfite sequencing (WGBS) was performed to screen enhancers with differential methylation. Then, CCAAT/enhancer-binding protein-beta (C/EBPβ) enhancer was selected for further function mechanism. The survival analysis indicated that hypomethylation of C/EBPβ enhancer was related to the poor prognosis of patients with HCC. The mechanism has been also investigated experimentally (Xiong et al., 2019). This study has inspired several studies to perform multi-omics analysis (Cui et al., 2021). By involving methylome, transcriptome, and 3D genomic data, the researchers comprehensively analyzed enhancer methylation regulome and identified enhancer methylation-enhancer TF-target gene expression. They found that the enhancer-regulated core TFs could further shape their enhancer methylation, thus forming the enhancer methylation-driven core transcriptional regulatory circuitries, which can be served as innovative therapy targets and prognostic risk biomarkers (Pan et al., 2022). In another study integrating ChIP-seq, RNA-seq, and WGBS data, the enhancers with differential expression and differential methylation were identified, as well as the associated differentially expressed genes. A model based on six enhancer-associated genes was constructed with regression analysis, exhibiting excellent predictive accuracy (Huang et al., 2022). In addition to screening biomarkers, the combination of epigenetic and transcriptional data can also demonstrate the mechanism. For example, the aberrant methylation of promoters and enhancers could activate critical cell cycle-related pathways and inhibit several metabolic pathways, thus affecting the progression of HCC (Huang et al., 2021). In short, the integrative analysis of multi-omics data can help us find new and more effective function targets in various diseases.

One comprehensive study has summarized more than 30 bioinformatics approaches for enhancer identification. With the advances in biological technologies, several data resources have been involved for screening enhancers, such as evolutionary conservation data, histone marks, Open chromatin, Transcription factor-binding sites, Sequencing features, Screening data, and eRNA expression. These data types can be combined in different ways to generate feature vectors that describe DNA regions. After feature selection, the feature vectors feed computational models that make decisions using unsupervised and/or supervised algorithms, such as Clustering, Classification, Graphical models, and Regression. The outcome is a list of identified enhancer regions. However, one of the major challenges is how to assess the correctness of predicted enhancers, because there is no large, sufficiently comprehensive, and experimentally validated enhancer set for humans. One possible way of validation is to link the predicted enhancers to their target genes (Kleftogiannis et al., 2016). The ELMER analysis applied in this study has involved five steps, which can construct a “TFs-motifs-genes” regulatory network. The formation of a regulatory network integrating TF, motif, and target gene further guaranteed the correctness of predicted candidates. However, one limitation of this study may be the lack of experimental evidence for validation. The prognostic prediction signature for LUAD has been only validated in patients from another two LUAD cohorts.

## Conclusion

This study has comprehensively analyzed the transcriptome and methylation data of a LUAD cohort. ELMER analysis has been performed to screen motifs, motif-associated TFs, and target genes. The “TFs-motifs-genes” regulatory network was constructed. After regression analysis, the Cox model involving eight genes was constructed. The enhancer-associated prognostic gene signature can be applied as a risk score for predicting the survival status of patients. With the increase of the risk scores, both the survival statuses and clinical traits showed a worse tendency in patients with lung adenocarcinoma. The multi-omics bioinformatics analysis can be a good tool for obtaining more information at the epigenetic level.

## Data Availability

The datasets presented in this study can be found in online repositories. The names of the repository/repositories and accession number(s) can be found below: www.ncbi.nlm.nih.gov/geo/, GSE31210, GSE8894.

## Supplementary material

Supplementary Material S1 131,371 distal probes selected with the get.feature.probe function.

Supplementary Material S2 10,665 distal hypomethylated probes identified with the get.diff.meth function in the tumor group.

Supplementary Material S3 669 pairs of statistically significant probe-gene pairs with negative correlations were screened by default parameters using the get.pair function in an unsupervised mode.

Supplementary Material S4 768 motifs enriched within the 250bp base sequence upstream and downstream of the probes in the 669 probe-gene pairs.

Supplementary Material S5 127 TFs with differential expressions in the two groups and negative correlations with methylation levels were screened by using the get.TFs function.

Supplementary Material S6 25 TFs and 80 target genes were selected as prognostic genes with the univariate analysis *p* value <0.05.
